# Antibacterial Efficacy and Mechanism of Mannosylerythritol Lipids-A on *Listeria monocytogenes*

**DOI:** 10.3390/molecules25204857

**Published:** 2020-10-21

**Authors:** Xiayu Liu, Qin Shu, Qihe Chen, Xinxin Pang, Yansha Wu, Wanyi Zhou, Yajing Wu, Jianrui Niu, Xinglin Zhang

**Affiliations:** 1 Department of Food Science and Nutrition, Zhejiang University, Yuhangtang Rd.866, Hangzhou 310058, China; Xiayuliu@zju.edu.cn (X.L.); 21713041@zju.edu.cn (Q.S.); chenqh@zju.edu.cn (Q.C.); 21713072@zju.edu.cn (X.P.); yanshawu@zju.edu.cn (Y.W.); munaiyi0612@zju.edu.cn (W.Z.); 21913063@zju.edu.cn (Y.W.); 2 College of Agriculture and Forestry, Linyi University, Linyi 276005, China

**Keywords:** Mannosylerythritol lipids-A (MEL-A), *Listeria monocytogenes*, antibacterial activity, RNA-Seq, milk, food-borne pathogen

## Abstract

Mannosylerythritol lipids-A (MEL-A) is a novel biosurfactant with excellent surface activity and potential biomedical applications. In this study, we explored the antibacterial activity and the underlying mechanisms of MEL-A against the important food-borne pathogen *Listeria monocytogenes*. The bacterial growth and survival assays revealed a remarkable antibacterial activity of MEL-A. Since MEL-A is a biosurfactant, we examined the cell membrane integrity and morphological changes of MEL-A-treated bacteria by biochemical assays and flow cytometry analysis and electron microscopes. The results showed obvious damaging effects of MEL-A on the cell membrane and morphology. To further explore the antibacterial mechanism of MEL-A, a transcriptome analysis was performed, which identified 528 differentially expressed genes (DEGs). Gene ontology (GO) analysis revealed that the gene categories of membrane, localization and transport were enriched among the DEGs, and the analysis of the Kyoto Encyclopedia of Genes and Genomes (KEGG) pathways demonstrated significant changes in the maltodextrin ABC transporter system and stress response system. Furthermore, the growth of *L. monocytogenes* could also be significantly inhibited by MEL-A in milk, a model of a real food system, suggesting that MEL-A could be potentially applied as an natural antimicrobial agent to control food-borne pathogens in the food industry.

## 1. Introduction

Food safety is one of the most important public health issues in the world. With the continuous development of globalization, the factors affecting food safety are becoming more and more diverse and complicated, and the incidence of food-borne diseases is constantly increasing. An analysis by the World Health Organization (WHO) finds that approximate 600 million people are infected with food-borne diseases each year, which causes more than 420,000 deaths [1]. Moreover, it is estimated that the real incidence of food-borne diseases is much higher than the reported incidence [2]. Among the numerous pathogenic factors, food-borne pathogens are major causes of food-borne diseases.

*L. monocytogenes* is a short rod-shaped Gram-positive bacterial and one of the most popular food-borne pathogens all over the world. In 1926, it was described for the first time in an outbreak which interfered with guinea pigs and rabbits [3] and recognized as a food-borne pathogen in 1980s [4]. In recent years, the number of people infected with listeriosis has constantly been rising and the mortality rate is extremely high (up to 20%–30%) [5]. Compared with healthy people, the mortality of susceptible people (elderly, children, pregnant women and immunocompromised individuals) is even higher. From the epidemiological data, there are three major types of *L. monocytogenes* (1/2a, 1/2b, and 4b) that are associated with human infections [6]. *L. monocytogenes* can survive in many extreme environments (low temperature, low pH, high salt concentration, etc.), so it is widely distributed in nature and easily causes food contamination in dairy products, seafood, meat, vegetables and ready-to-eat food [7]. Hence, *L. monocytogenes* is a continuous threat to the food industry due to its high lethality, and its intrinsic and acquired tolerance to commonly used antimicrobial agents. Therefore, there is great demand for novel reagents (especially natural food preservatives) that can prevent *L. monocytogenes* contamination in food industry.

Biosurfactants are an important class of surfactants, which can be produced by microorganisms using sugar, oil, etc. as substrates. According to different structures, biosurfactants can be divided into glycolipids, lipopeptides and lipoproteins, phospholipids, fatty acids and polymeric biosurfactants [8,9]. Glycolipids (including rhamnolipids, sophorolipids, trehalolipids, etc.) are considered as among the most important types of biological surfactants because of their excellent antibacterial activity against common pathogens. Mannosylerythritol lipids (MELs) are a representative type of glycolipids, which not only exhibit great emulsification, surface activity and biodegradability, but also have the functions of inhibiting microbial growth, inducing cell differentiation, and improving gene transfection efficiency [10]. Based on the difference of the fatty acid chain and acetyl group, MELs can be divided into four different configurations: MEL-A, MEL-B, MEL-C, and MEL-D [11]. Four different MELs can be produced by different microorganisms through different metabolic pathways. In our previous research, MELs showed outstanding antibacterial effects against *Staphylococcus aureus* [12] and *Bacillus cereus* [13]. Among the four different MELs, MEL-A has a simpler production route and more efficient antibacterial properties. To date, however, there has been little discussion about the inhibitory effect of MEL-A on *L. monocytogenes*.

The goal of this work was to study the antibacterial activity and the mechanism of MEL-A against *L. monocytogenes*, through biochemical analyses, morphology observation, transcriptome profiling and other methods. Furthermore, milk was used as a model system to investigate the antibacterial effect of MEL-A in complex food systems, so as to explore the potential application of MEL-A in the food industry. In this study, we found that MEL-A has an excellent inhibitory effect on *L. monocytogenes* by causing damages on the cell membrane. Through transcriptome analysis, we discovered that MEL-A could lead to the dramatic reprograming of gene expression, particularly for the ABC transporter systems and other important pathways mainly involved in stress response.

## 2. Materials and Methods

### 2.1. Microorganisms and Chemicals

The bacterial *L. monocytogenes* wild-type strain EGD-e (ATCC BAA-679) was obtained from American Tissue Culture Collection and maintained in slants of brain–heart infusion (BHI) agar or broth at 4 °C. Other strains were stored in our laboratory. Pasteurization-processed skimmed milk and whole milk were purchased from a local grocery store (Wal-mart Superstore, Hangzhou, China). All the chemicals used in this study were of analytical grade.

### 2.2. Production and Purification of MEL-A

MEL-A was produced and purified as previously reported by Fan et al. [14]. After 7 days of fermentation, MELs were produced from vegetable oil by *Pseudozyma aphidis* DSM 70,725 and then mixed with the same volume of ethyl acetate. The organic layer was separated by extraction, and the ethyl acetate was evaporated under reduced pressure. Then, the crude MELS were washed twice with methanol and cyclohexane to remove the residual oil and fatty acids, which were also purified through a silica gel column and then analyzed by thin layer chromatography (TLC) (Silica gel 60F, chloroform:methanol:water = 70:15:2, *v*/*v*), liquid chromatography-mass spectrometry (LC-MS) (Agilent, Sacramento, CA, USA), gas chromatography-mass spectrometry (GC-MS) (Hiden, London, UK) and nuclear magnetic resonance (NMR) (Thermo Fisher Scientific, Waltham, MA, USA) [15]. Experimental results showed that the MEL-A accounts for more than 80% of the MELS.

### 2.3. Production and Purification of MEL-A

#### 2.3.1. Determination of Minimum Inhibition Concentration (MIC) in BHI

MIC is one of the most common indicators for detecting the lowest concentration of antimicrobial agent that prevents the visible cell growth of microorganisms under specific conditions [16]. MEL-A were added to BHI broth by two-fold serial dilution method, the final concentrations were 0, 4, 8, 16, 32, 64, and 128 μg/mL, respectively. Fifty milliliters of *L. monocytogenes* was cultured at 37 °C in BHI until the exponential phase, and then washed three times with phosphate buffer saline (PBS) and diluted to obtain a final concentration of 1.0 × 10^6^ CFU/mL as the seed. Then, 1 mL of *L. monocytogenes* was inoculated into a 50 mL reagent bottle containing sterile BHI with different concentrations of MEL-A, and all the flasks were incubated in an orbital shaker (37 °C, 180 rpm) (Thermo Fisher Scientific, Waltham, MA, USA). After remaining overnight, the bacterial concentration was taken out and monitored by measuring the OD_600nm_ value using a microplate reader (Thermo US).

#### 2.3.2. Bacterial Growth Curve in BHI

To assess the inhibitory effect of different MEL-A concentrations on different growth stages of *L. monocytogenes*, the bacterial growth curve was drawn. After preparing the BHI broth with different concentrations of MEL-A (0, 16, 32, 64,128 μg/mL), the logarithmic phase of *L. monocytogenes* was inoculated into shake flasks and cultured at 37 °C. Then, 100 μL of bacterial suspension was taken out from the flask every 2 h, diluted and spread evenly on the BHI plate, and incubated in a 37 °C incubator. The growth curve of *L. monocytogenes* was drawn using the plate counting method and expressed as a log CFU/mL sample [17].

#### 2.3.3. Bacterial Survival Rate

Cell Counting Kit-8 (Beyotime Biotechnology, Shanghai, China) was a rapid and highly sensitive detection kit based on WST-8 (2-(2-methoxy-4-nitrophenyl)-3-(4-nitrophenyl)-5-(2,4-disulfophenyl)-2*H*-tetrazolium, monosodium salt) and widely used in cell proliferation and cytotoxicity detection [18]. It was used to further determine the survival rate of *L. monocytogenes* treated with MEL-A. The BHI broth was prepared with different concentrations of MEL-A (0.5 × MIC, 1 × MIC, 2 × MIC), and then 1 mL of *L. monocytogenes* (1.0 × 10^6^ CFU/mL) in the logarithmic growth phase was inoculated into each reagent bottle and cultured at 37 °C for 12 h. Then, the bacterial cultures were diluted 100 times. According to the kit instructions, 180 μL of diluent and 20 μL CCK-8 solution were added to a 96-well plate and mix gently. After incubating in a cell incubator at 37 °C for 0.5, 1 and 2 h, the values of OD_450_ and OD_650_ were measured with a microplate reader (Thermo Fisher Scientific, Waltham, MA, USA). The survival rate of *L. monocytogenes* was calculated by the following formula (1):

S = 100% × (M(OD_450_) − M(OD_650_))/(C(OD_450_) − C(OD_650_)) (1)

S: survival rate; M: MEL-A treated; C: control.

### 2.4. Antibacterial Mechanism of MEL-A

#### 2.4.1. The Integrity of Cell Membrane Assays

To determine the integrity of cell membrane, the leakage of intracellular constituents including nucleic acids and proteins was measured as described by the literature [19]. As in the method described above, the *L. monocytogenes* was inoculated into different concentrations of MEL-A (0.5 × MIC and 1 × MIC) and cultured at 37 °C and 180 rpm. After 12 h, the bacterial suspension was taken out and centrifuged at 8000 rpm for 3 min, and then the supernatants were filtered by a 0.22 μm membrane. To determine the concentrations of the leakage of intracellular constituents that consist of nucleic acids and proteins, the absorbance of the supernatants was measured at 260 nm and 280 nm by a UV-visible spectrophotometer (Agilent, Sacramento, CA, USA).

#### 2.4.2. Flow Cytometric Analysis

Propidium iodide (PI) is a nucleic acid dye which cannot penetrate the complete cell membrane of normal cells or early apoptotic cells but can stain the nucleus red through the cell membrane of late apoptotic and necrotic cells [20]. Thus, it was used to distinguish the survival early cells from necrotic or late apoptotic cells. The suspensions of *L. monocytogenes* in BHI broth (treated with MEL-A at the concentration of 0, 0.5 × MIC, 1 × MIC, and 2 × MIC) were incubated at 37 °C, 180 rpm for 12 h as above. Then, the cells were washed twice with cold PBS and gently mixed 1–5 × 10^5^ cells with 10 μL PI staining solution. After that, the mixture was incubated at room temperature in the dark for 10–15 min. The samples were monitored by flow cytometric (FACSVerse, Newark, NJ, USA) and the data were analyzed by FlowJo 10.

#### 2.4.3. Morphological Observation of *L. monocytogenes*

*L. monocytogenes* was cultured in BHI (treated with MEL-A at the MIC) at 37 °C for 12 h, while the bacterial cells without MEL-A were used as the control. The samples were washed with PBS and 2.5% glutaraldehyde at 4 °C overnight. Then, the bacterial was observed by SEM (Hitachi SU-8010, Tokyo, Japan) and TEM (Hitachi H-7650, Tokyo, Japan) in the bio-ultrastructure analysis Lab of Analysis center of Agrobiology and environmental science (Zhejiang University).

#### 2.4.4. RNA Sequencing

Approximately 1 × 10^6^ CFU of *L. monocytogenes* were inoculated into 50 mL of BHI broth (treated with MEL-A at the MIC) and the bacterial cells without MEL-A was used as the control, and grown at 37 °C until the exponential phase. The bacterial solutions were centrifuged at room temperature (5 min; 4000 rpm) and were quickly frozen in liquid N_2_ as described previously [21]. Strand-specific libraries were generated using NEBNext^®^ UltraTM RNA Library Prep Kit (New England Biolabs, Los Angeles, CA, USA), then the library preparations were sequenced on an Illumina Novaseq platform and 150 bp paired-end reads were generated. Then, the quantification of the gene expression level was analyzed by HTSeq v0.6.1 (New South Wales, Sydney) and the differential expression analysis of two conditions was performed using the DESeq R package (1.18.0) (New South Wales, Sydney). Gene ontology (GO) enrichment analysis of differentially expressed genes was implemented by the GOseq R package, and the KOBAS software (Beijing, China) was used to test the statistical enrichment of the differential expression genes in the Kyoto Encyclopedia of Genes and Genomes (KEGG) pathways.

#### 2.4.5. Real-Time PCR Analysis

Total RNA was isolated as described above and cDNA was synthesized with the PrimeScript RT reagent Kit (Thermo Fisher Scientific, Waltham, MA, USA) with the gDNA Eraser. Real-time PCR on this cDNA was performed using the TB Green^®^ Premix Ex Taq™ (Tli RNaseH Plus) Kit (Thermo Fisher Scientific, Waltham, MA, USA) and an Applied Biosystems™ QuantStudio™ 3 instrument (Thermo Fisher Scientific, Waltham, Massachusetts, USA). The expression of *drm* was used as a housekeeping control. The QuantStudioTM Design & Analysis Software 1.3.1 (Thermo Fisher Scientific, Waltham, MA, USA) was used to calculate the Ct values and the relative gene expression was calculated using the 2^−ΔΔCt^ method [22].

### 2.5. Inhibition of Bacterial Growth in Milk by MEL-A

The bacterial growth curve was drawn to determine the antibacterial effect of MEL-A against *L. monocytogenes* in whole milk and skimmed milk. Approximately 1 × 10^6^ CFU of *L. monocytogenes* were inoculated into 25 mL of milk with different concentrations of MEL-A (0 μg/mL, 512 μg/mL, 1024 μg/mL) and incubated statically at 37 °C. Then, the plate counting method described above was used to count the colonies of each sample every 12 h.

### 2.6. Statistical Analysis

All experiments were performed with three biological replicates and the data were presented as the mean ± standard deviation. All significant differences were carried out using a one-way analysis of variance (ANOVA) and Duncan’s multiple range test (DMRT). And all the tests were considered statistically significant only when *p* < 0.05.

## 3. Results and Discussions

### 3.1. Inhibition Effect of MEL-A against L. monocytogenes

#### 3.1.1. MIC of MEL-A against *L. monocytogenes*

The minimum inhibitory concentration (MIC) was designed to determine the antimicrobial activity of MEL-A on *L. monocytogenes*. As we can see from Figure S1, there was a significant inhibitory effect of MEL-A when the concentration reached 32 μg/mL, which was regarded as the MIC. This finding was in agreement with previous findings [23], showing the strong antibacterial activity of MEL-A. In addition, compared with the MIC of MELs against *B. cereus* [13], our results also indicated that the pure substance MEL-A had a better antibacterial effect than the mixture of MELs.

#### 3.1.2. Effect on Bacterial Growth

**Figure 1:**
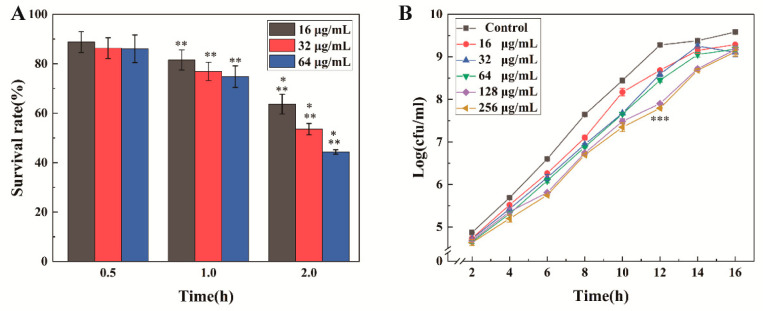
The effect of mannosylerythritol lipids-A (MEL-A) on bacterial growth. (**A**): survival rate of *L. monocytogenes* treated with MEL-A; (**B**): the effect of MEL-A on the *L. monocytogenes* growth curve. * (one asterisk) indicates a significant difference in the comparison between different concentrations at the same time. ** (two asterisks) indicate a significant difference in the comparison between the same concentration at different times. *** (three asterisks) indicates that the 128 μg/mL treatment group is significantly different from the control group.

In order to further confirm the antibacterial activity of MEL-A against *L. monocytogenes*, the Cell Counting Kit-8 was used to detect the number of living cells [18,24]. In the presence of electronic coupling reagents, WST-8 in the Cell Counting Kit-8 (CCK-8) can be reduced to orange formazan and the color intensity has a linear relationship with the number of cells. Therefore, we can judge the number of living cells in different MEL-A treatment groups by the absorbance value. The living cells continuously decreased with the increase in MEL-A concentration and the extension of culture time (Figure 1A). After two hours of incubation, only 63.65% (0.5 × MIC), 52.57% (1 × MIC) and 44.30% (2 × MIC) of *L. monocytogenes* survived.

Bacterial growth assay showed that the MEL-A had an inhibition effect on *L. monocytogenes* in the logarithmic growth phase (Figure 1B). The growth curve of *L. monocytogenes* treated with MIC (32 μg/mL) was approximately 1 log lower than that of the control group from 8 to 12 h. When the concentration of MEL-A reached 128 μg/mL, the experimental group was approximately 1.5 log lower than the control group at 12 h. In summary, these results indicated the excellent inhibitory effect of MEL-A on *L. monocytogenes*.

### 3.2. The Effect of MEL-A on the Integrity of Cell Membrane

**Figure 2:**
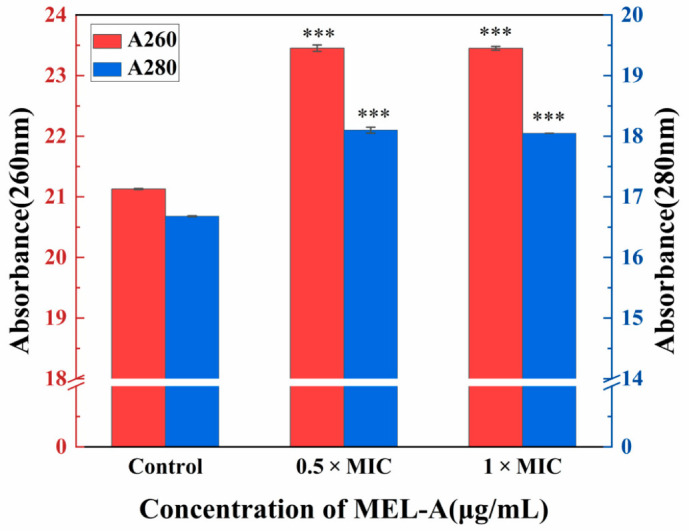
Leakage of the intracellular nucleic acids and proteins. *** (three asterisks) represents significant differences (*p* < 0.001) compared to controls. All experiments were performed with three biological replicates (*n* = 3).

In order to confirm the possible impact of MEL-A on the cell membrane of *L. monocytogenes*, the leakage of nucleic acids and proteins which accounted for a large proportion of intracellular substance were measured [25]. As we can see from Figure 2, there was a significant difference in the absorbance at 260 nm (nucleic acids) and 280 nm (proteins) between the experimental groups and the control group. Therefore, the inhibitory effect of MEL-A on *L. monocytogenes* could be attributed to the damage to cell membrane. These findings were in agreement with several previous studies that investigated the inhibitory mechanism of antibacterial substances against *L. monocytogenes* [26,27,28].

### 3.3. Flow Cytometric Analysis

**Figure 3:**
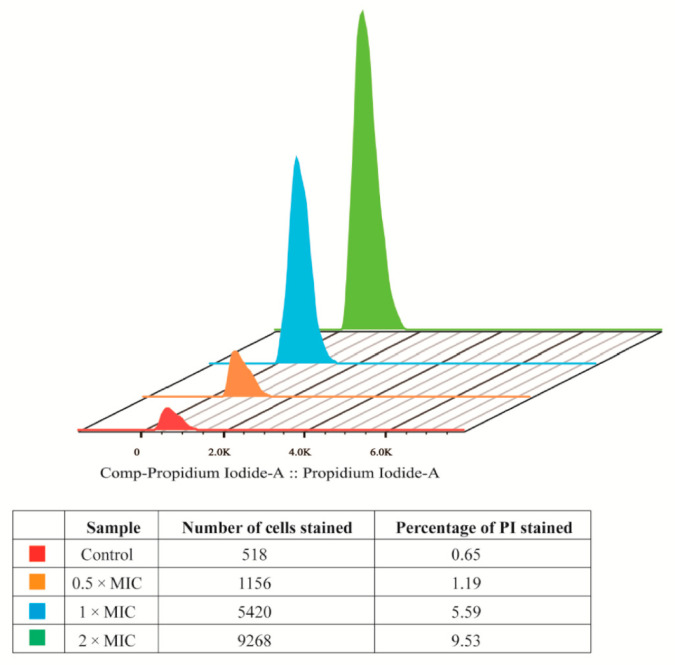
Fluorescence plots of *L. monocytogenes* stained with propidium iodide (PI).

As one of the most advanced cell quantitative analysis techniques, Flow cytometry (FCM) has become widely used due to its fast speed, high precision and accuracy. PI (nucleic acid dye) cannot permeate the normal cells with complete cell membrane but can dye the nucleus red in the late apoptotic cells and necrotic cells with incomplete membrane. Hence, to detect the cell viability and the integrity of cell membrane more precise, the fluorescent staining with PI was employed [29]. Figure 3 shows that the fluorescence signal stained by PI in the MELA-treated group was much higher than the control. The difference between different concentrations (0.5 × MIC, 1 × MIC, 2 × MIC) was also apparent. From the table below we can see that the proportion of late apoptosis cells and necrotic cell is 1.19%, 5.59%, and 9.53% after treating with MEL-A at level of 0.5 × MIC, 1 × MIC, and 2 × MIC, respectively, which was significantly more than 0.65% in the control group. In summary, these findings suggest that the destruction of cell membrane by MEL-A may be the main cause of the decrease in cell viability, which is also consistent with the results above.

### 3.4. Morphological Analysis of L. monocytogenes

**Figure 4:**
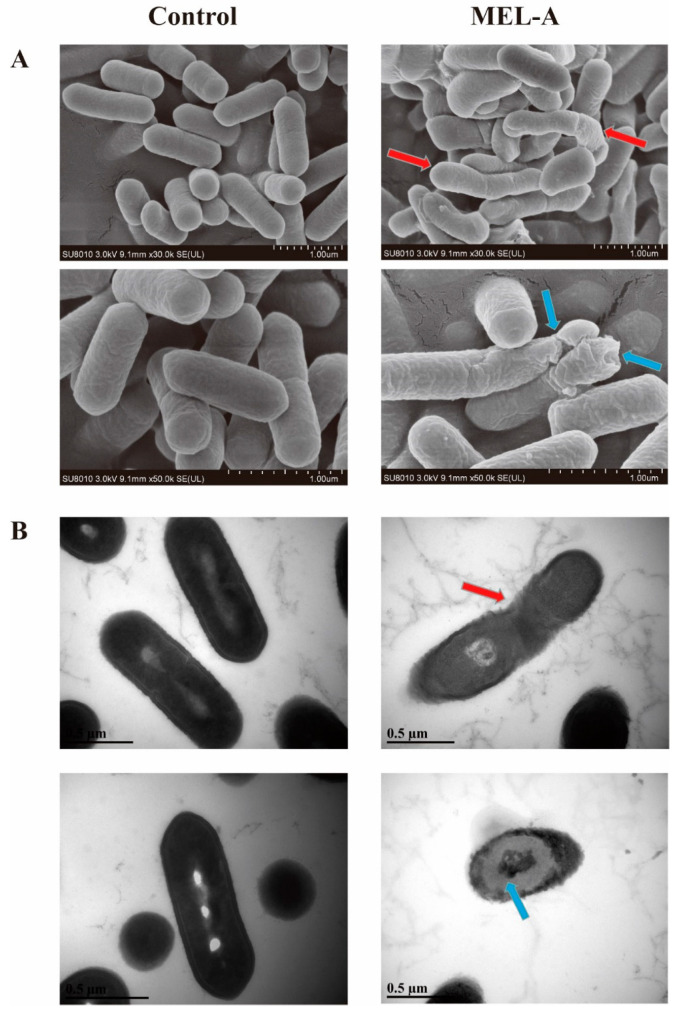
SEM (**A**) and TEM (**B**) micrographs of *L. monocytogenes* treated with MEL-A at the minimum inhibitory concentration (MIC) and control cells without treatment.

SEM was used to observe the external morphology changes of the *L. monocytogenes* cells [30]. The SEM images (Figure 4A) of the MEL-A-treated cells were significantly different to those of the untreated cells. The cells in the control group were short rod-shaped and plump, and the surface structure was smooth and flat. Conversely, the *L. monocytogenes* cells treated with MEL-A became slender and irregular (red arrows), and the surface roughness increased significantly. Even some holes were observed in the cytoplasmic membranes as shown by the blue arrows. These results visually show the destruction of MEL-A to the external structures of *L. monocytogenes*.

To further observe the external or internal cell structures changes caused by MEL-A, treated and untreated *L. monocytogenes* cells were examined by TEM. As the picture shows in Figure 4B, the cells in the control appeared normal with intact cell membranes and a dense cytoplasm. In contrast, *L. monocytogenes* with MEL-A treatment showed irregular shape with the sunken surfaces (red arrow), and the cross section was much shallower than the control (blue arrow). These results suggested that MEL-A had a stronger impact on the cell membrane and the leakage of intracellular contents including nucleic acids and proteins, which was similar to the effect of carvacrol on *L. monocytogenes* [30]. In conclusion, the morphological observation of *L. monocytogenes* provides compelling evidence that MEL-A is able to damage the cell membrane and eventually result in the leakage of intracellular substances and cell death.

### 3.5. Transcriptome Analysis

#### 3.5.1. Analysis of the Differentially Expressed Genes (DEGs)

**Figure 5:**
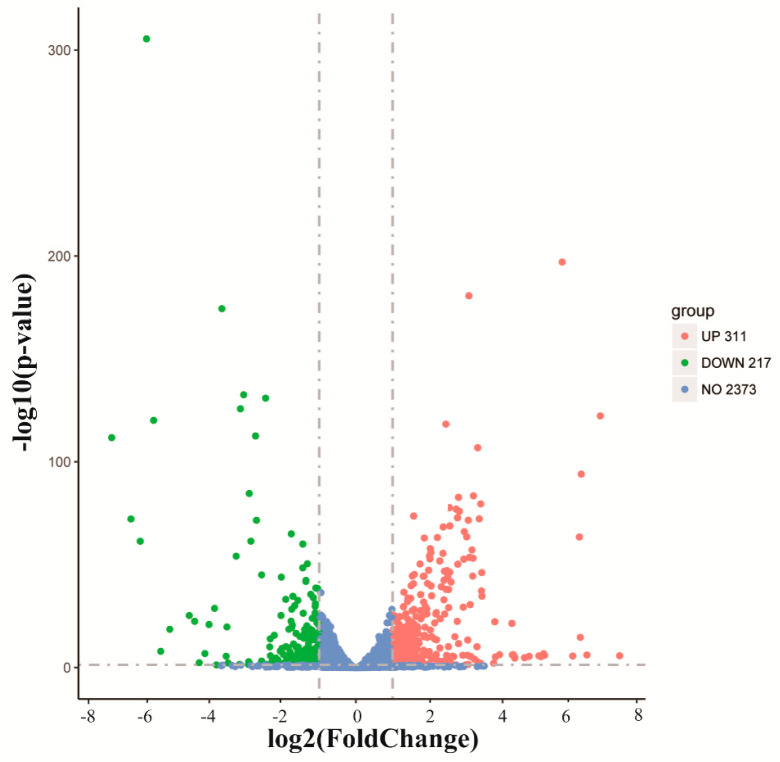
Analysis of deferentially expressed genes. The transcription data from the MEL-A-treated cells were compared to those from the control condition. The genes with a *p*-value < 0.05 and |log2(FoldChange)| > 1 were considered differentially expressed genes (DEGs). Red dots indicate upregulated DEGs, green dots indicate downregulated DEGs and the blue dots indicate genes with no significant changes in expression.

Transcriptome analysis was applied to further elucidate the antibacterial mechanism of MEL-A. After MEL-A treatment, 528 differentially expressed genes were identified, including 311 upregulated and 217 downregulated genes (Figure 5). Within the upregulated genes, 28.3% of the genes with fold change (FC) > 5, 5.5% of the genes with FC > 20, and there were three genes (lmo2180, lmo0481, lmo2336) with FC > 100. Within the downregulated genes, 18% of the genes with FC > 5, 4.6% of the genes with FC > 20, and there were two genes (lmo2125, lmo2124) with FC > 100. Overall, these results suggested that there was a significant difference between the MEL-A treatment group and the control group.

#### 3.5.2. Gene Ontology (GO) Analysis

**Figure 6:**
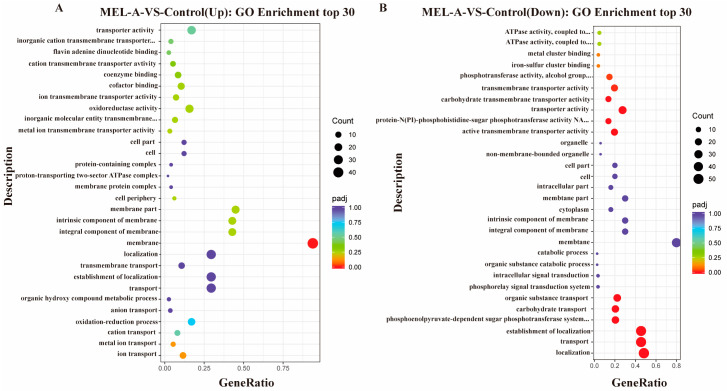
Scatter plot of gene ontology (GO) enrichment analysis: (**A**) scatter plot of the upregulated genes; and (**B**) scatter plot of the downregulated genes. The cut off value was screened based on *p* < 0.05 and |log2(FC)| > 1. The abscissa is the ratio of the number of differential genes annotated to GO Term to the total number of differential genes, and the ordinate is the GO Term.

The top thirty upregulated and downregulated genes by GO enrichment analysis are presented in Figure 6. The expression of genes involved in membrane localization and transport were significantly more regulated by the exposure to MEL-A (Table S1). After MEL-A treatment, the expression of the membrane-associated gene (membrane, membrane part and integral component of membrane) were upregulated (Figure 6A), which could be a protective response of bacteria to the stress caused by MEL-A [31]. The expression of genes involved in localization (lmo2124, lmo1740, etc.), transport (lmo2124, lmo2123, lmo2347, etc.) and the establishment of localization were downregulated (Figure 6B). These genes are mainly predicted to be related to the transport of sugar and amino acids, indicating that MEL-A directly affected the utilization of carbohydrates and the biosynthesis of amino acids, further affecting the ability of bacteria to repair themselves in extreme environments. This might be an important mechanism for MEL-A to inhibit bacterial growth.

#### 3.5.3. KEGG Analysis

**Figure 7:**
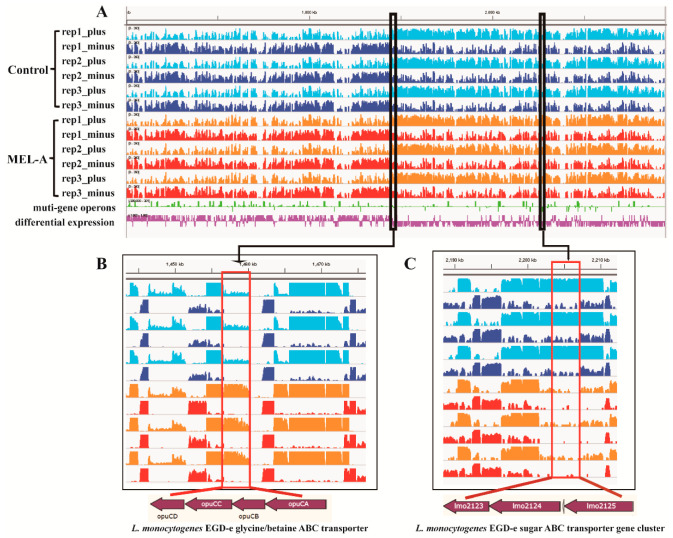
Transcriptome analysis of *L. monocytogenes*: (**A**) coverage plots of RNA-seq data aligning to chromosome DNA. The *y* axis represents the reads coverage while the *x* axis denotes the genomic location. Light blue (control) or orange (MEL-A) tracks are reads aligned to the plus strand and blue (control) or red (serum) tracks are reads aligned to the minus strand. Multi-gene operons are shown in the green track and differentially expressed genes are expressed in purple track. (**B**) An upregulated gene cluster related to glycine/betaine ABC transporter. (**C**) A downregulated gene cluster related to sugar ABC transporter.

KEGG (Kyoto Encyclopedia of Genes and Genomes) is a comprehensive database that integrates the genome, chemistry and system function information. Altogether, five pathways (Table S2) were differentially regulated during cultivation in MEL-A, in which the ABC transporters-associated genes (lmo2123, lmo2123, opuCD, etc.) were significantly regulated. ABC transporters belong to a large and diverse protein family that are mainly responsible for transporting various substrates (including ions and macromolecules) and are functionally important for the occurrence and functional maintenance of the membrane [32]. By MEL-A treatment, 14 genes related to ABC transporters were upregulated and 36 genes were downregulated. Among the most differentially expressed genes, we detected a significantly upregulated gene cluster (Figure 7B) with an important role in glycine/betaine transport and a downregulated gene cluster (Figure 7C) with an important role in sugar transport.

A previous study has shown that the opuC operon (including OpuCA, OpuCB, OpuCC, and OpuCD) plays an important role in osmoregulation in *L. monocytogenes* [33]. Therefore, under the treatment of MEL-A, the genes related to opuC operon are upregulated to maintain a stable osmotic pressure, which is consistent with the findings in *Bacillus subtilis* [34] and *Staphylococcus aureus* [35]. In addition, the glycine/betaine transport system was also found to contribute to bile (causing membrane damage) resistance in *Enterococcus faecium* [36]. Furthermore, the downregulated gene cluster (lmo2123–lmo2125) has been predicted to play an important role in encoding components of a maltodextrin ABC transporter system [37,38]. This might be an adaptive response of *L. monocytogenes* to MEL-A by shutting down certain transporters to reduce the bioactivity and permeability of cell membrane. Hence, the interference on bacterial transporter systems might be one of the antibacterial mechanisms of MEL-A.

### 3.6. Real-Time PCR Analysis

**Figure 8:**
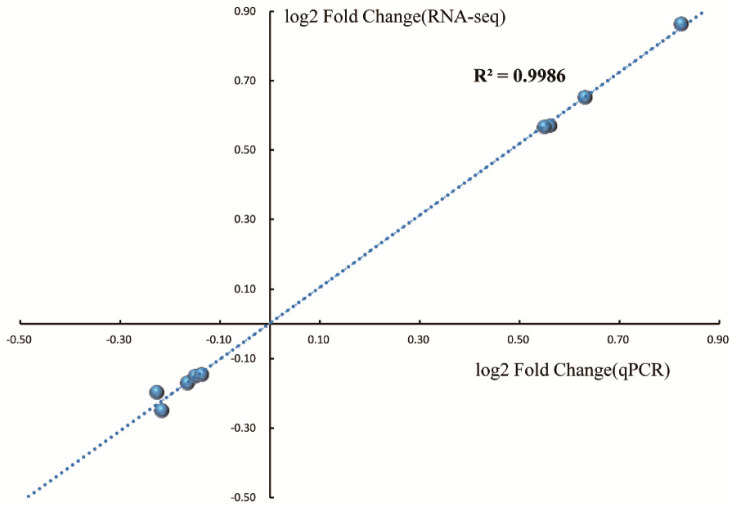
RT-qPCR validation of RNA-Seq experiments. The gene expression ratios obtained from both qPCR and RNA-Seq were normalized by a housekeeping control gene *drm*. Each group was performed with three biological replicates.

In order to confirm the RNA-Seq data, we analyzed the transcription levels of nine genes (Table S3) during growth in MEL-A treated BHI and untreated BHI by qPCR. As shown in Figure 8, the results of RNA-Seq and qPCR were highly consistent (r^2^ =  0.9986).

### 3.7. Antimicrobial Activity of MEL-A in Milk

**Figure 9:**
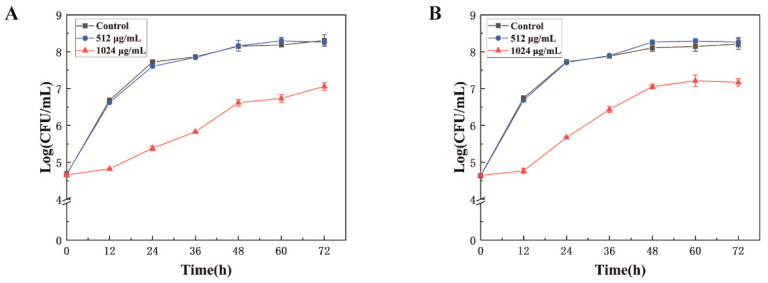
Effect of MEL-A on the growth of *L. monocytogenes* in whole milk (**A**) and skimmed milk (**B**) at room temperature (25 °C). Error bars are standard deviations (*n* = 3).

Milk was use as a model to examine the inhibitory effect of MEL-A on *L. monocytogenes* in real food systems. As we can see from Figure 9, when the concentration of MEL-A reached 1024 μg/mL, there was an obvious inhibitory effect on the growth of *L. monocytogenes* in milk. The growth curve was almost 2 log lower than that of the control group at 12 h and 24 h. Nevertheless, no significant differences were found between the MEL-A-treated at 512 μg/mL and the control group. A likely explanation is that some complex food ingredients interfere with the function of MEL-A as previous findings [39]. Similarly, comparing Figure 9A with Figure 9B, there was no significant difference in the application of MEL-A in whole milk and skimmed milk. This finding indicates that MEL-A was not affected by the fat content in milk, showing its superiority over other antibacterial substances [17]. However, compared with the MIC values recorded in the culture media (32 μg/mL), the MIC value in milk (1024 μg/mL) was much higher. It may be attributed to the complexity of milk composition, since milk is rich in biomolecules and other components that may bind to MEL-A and interfere with its activity. Although the MIC in milk is relatively higher than that under experimental conditions, MEL-A could significantly inhibit the growth of *L. monocytogenes* in the real food system, implicating potential application prospects in the food industry by the further optimization or combination with other antibacterial substances.

## 4. Conclusions

In conclusion, this study identified and evaluated the bacteriostatic and bactericidal effect of MEL-A, on the important food-borne pathogen *L. monocytogenes*. This antibacterial activity is mainly attributed to the destruction of cell membrane and the impact on the ABC transport systems and stress response systems. In addition, *L. monocytogenes* could also be significantly inhibited by MEL-A when grown in milk. Although a relative high concentration is required in such a food model that may contain biomolecules and other components interfering with the activity of MEL-A, by further optimizing or combining with other antibacterial substances, this naturally produced and highly productive antibacterial agent could potentially be applied to the food industry for the control of food-borne pathogens.

## Supplementary Materials

The following are available online. Figure S1: Inhibition effect of different concentrations of MEL-A on *L. monocytogenes* and other strains, Table S1: Fold change of major differentially expressed genes in Go enrichment analysis, Table S2: KEGG analysis, Table S3: qRT-PCR validation of RNA-seq experiments.
