# Supplementary material for: Antibacterial Efficacy and Mechanism of Mannosylerythritol Lipids-A on Listeria monocytogenes
Source: Molecules. 2020 Oct 21;25(20):4857. doi: 10.3390/molecules25204857 (PMC7587930; doi:10.3390/molecules25204857)
Supplement: Supplementary file 1 [file molecules-25-04857-s001.pdf]

Molecules

Antibacterial efficacy and mechanism of mannosylerythritol lipids-A on *Listeria monocytogenes* based on transcriptional analysis

Xiayu Liu [a], Qin Shu [a], Qihe Chen [a] , Xinxin Pang [a], Yansha Wu [a], Wanyi Zhou [a], Yajing Wu [a], Jianrui Niu [b,*], Xinglin Zhang [a,b,*]

[a] *Department of Food Science and Nutrition, Zhejiang University, Hangzhou 310058, China*

[b] *College of Agriculture and Forestry, Linyi University, Linyi 276005, China*

****Corresponding author:***

Xinglin Zhang: xinglinzhang@zju.edu.cn

Jianrui Niu: niujianrui@lyu.edu.cn

Yuhangtang Rd.866

Department of Food Science and Nutrition

Zhejiang University

Hangzhou 310058

P.R.China

Tel: +86-571-86984316

Supplementary file

Figure S1 Inhibition effect of different concentrations of MEL-A on *L. monocytogenes* and other strains.

Table S1 Fold change of major differentially expressed genes in Go enrichment analysis.

Table S2 KEGG analysis.

Table S3 qRT-PCR validation of RNA-seq experiments.

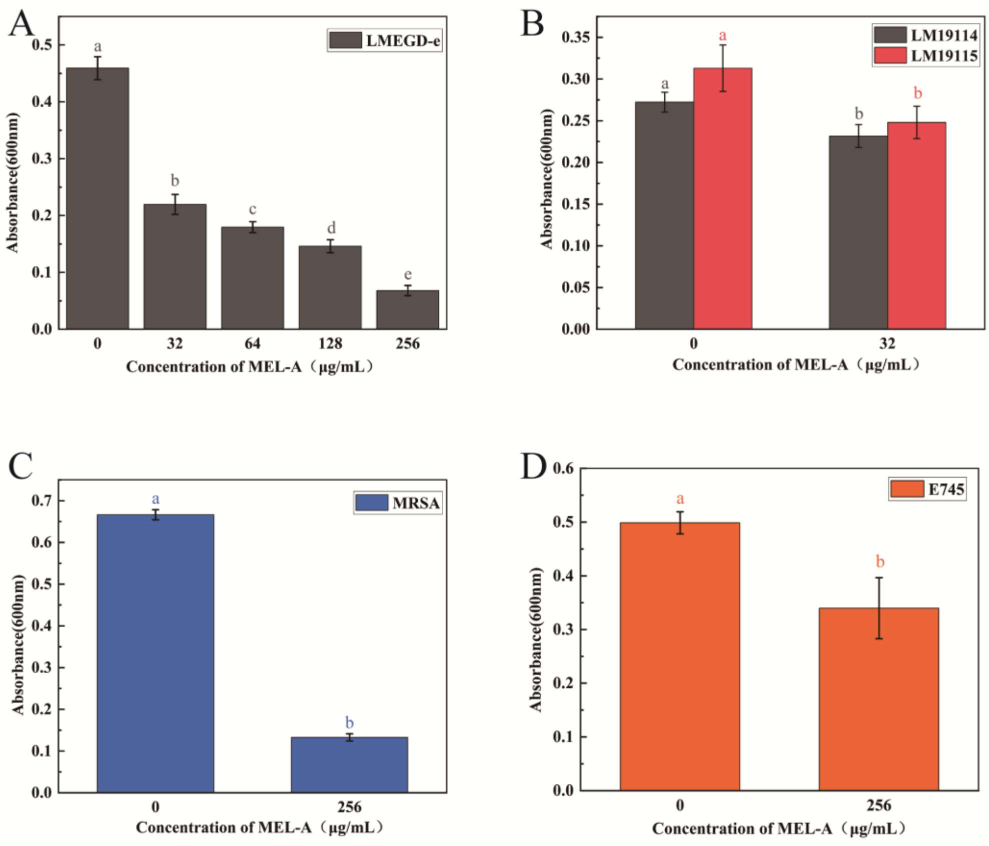

Fig. S1. Inhibition effect of different concentrations of MEL-A on *L. monocytogenes* and other strains. (A) *L. monocytogenes* wild-type strain EGD-e (B) LM19114 represents *L. monocytogenes* 19114, and LM19115 represents *L. monocytogenes* 19115. (C) MRSA represents methicillin-resistant *Staphylococcus aureus* (D) E745 represents *Enterococcus faecium* E745, Bacteria were grown under different concentrations of MEL-A at 37 ℃ for 12 hours. Then the absorbance 600 nm were measured. The different letters within the same indicator indicate a significant difference between the concentrations ($p < 0.05$).

Table S1 Fold change of major differentially expressed genes in Go enrichment analysis.

Up/Down	Description	gene_id	FoldChange
UP	membrane	lmo2335	49.35
		lmo2387	7.68
		lmo1526	3.36
		lmo0723	5.59
		lmo2697	9.09
Down	localization	lmo2124	0.01
		lmo1740	0.13
	transport	lmo2124	0.01
		lmo2123	0.02
		lmo2347	0.03
	establishment of localization	lmo2124	0.01
		lmo2123	0.02
		lmo0097	0.37

Table S2 KEGG analysis.

KEGGID	Description	pvalue	Count	Up	Up_Gene_names	Down	Down_Gene_names
lmo00220	Arginine biosynthesis	0.002075	12	10	lmo0043/lmo2090/lmo2091/lmo1591/lmo1590/lmo1299/lmo1588/lmo0039/lmo1589/lmo1587	2	lmo0560/lmo1897
lmo01210	2-Oxocarboxylic acid metabolism	0.002733	18	12	lmo1591/lmo1984/lmo1590/lmo1986/lmo1588/lmo1589/lmo1987/lmo1983/lmo1985/lmo1988/lmo1989/lmo1567	6	lmo1436/lmo1235/lmo1437/lmo2374/lmo1897/lmo0978
lmo02024	Quorum sensing	0.020296	29	8	lmo0539/lmo2434/lmo2515/lmo1295/lmo2569/lmo2612/lmo0037/lmo0448	21	lmo0152/lmo0135/lmo0137/lmo1529/lmo0202/lmo0136/lmo0051/lmo1803/lmo2854/lmo2196/lmo0205/lmo0269/lmo0245/lmo2451/lmo0048/lmo2195/lmo2193/lmo2194/lmo1210/lmo1801/lmo2192
lmo01230	Biosynthesis of amino acids	0.026893	75	32	lmo0265/lmo2205/lmo2090/lmo2091/lmo2674/lmo1591/lmo2458/lmo2459/lmo1984/lmo1590/lmo1986/lmo1299/lmo2457/lmo1588/lmo1305/lmo1749/lmo1589/lmo1987/lmo1983/lmo2456/lmo1011/lmo1571/lmo1985/lmo1988/lmo1989/lmo1587/lmo0359/lmo1567/lmo0342/lmo1620/lmo2455/lmo0343	43	lmo0223/lmo2545/lmo2546/lmo0238/lmo1259/lmo1072/lmo2547/lmo1436/lmo1536/lmo1813/lmo1435/lmo1235/lmo1437/lmo1678/lmo1924/lmo2824/lmo2374/lmo1923/lmo1490/lmo0199/lmo0491/lmo2660/lmo0490/lmo1907/lmo2236/lmo1925/lmo1681/lmo1627/lmo1600/lmo1631/lmo0570/lmo0594/lmo1897/lmo2370/lmo0978/lmo1680/lmo2659/lmo0564/lmo1818/lmo2661/lmo1630/lmo2539/lmo1679
lmo02010	ABC transporters	0.029872	50	14	lmo1426/lmo2250/lmo1425/lmo1427/lmo1428/lmo2745/lmo1421/lmo2251/lmo0919/lmo2495/lmo1014/lmo1422/lmo0847/lmo0848	36	lmo2125/lmo2124/lmo2123/lmo2349/lmo2417/lmo2346/lmo2348/lmo0283/lmo2347/lmo0284/lmo1390/lmo1671/lmo2196/lmo1446/lmo1391/lmo1388/lmo2418/lmo2419/lmo1848/lmo1389/lmo0285/lmo2599/lmo1849/lmo0987/lmo1847/lmo2634/lmo1041/lmo2195/lmo2600/lmo2193/lmo2715/lmo2194/lmo2007/lmo2192/lmo2506/lmo0153

Table S3 qRT-PCR validation of RNA-seq experiments.

gene_id	FoldChange(qPCR)	FoldChange(RNA-seq)
lmo1425	3.447	3.374
lmo1426	3.532	3.397
lmo1427	3.001	2.893
lmo1428	2.321	2.231
lmo2123	0.015	0.017
lmo2124	0.010	0.010
lmo2125	0.006	0.009
lmo2347	0.047	0.030
lmo2348	0.041	0.062
